# Establishment of feeder-free culture system for human induced pluripotent stem cell on DAS nanocrystalline graphene

**DOI:** 10.1038/srep20708

**Published:** 2016-02-05

**Authors:** Hyunah Lee, Donggyu Nam, Jae-Kyung Choi, Marcos J. Araúzo-Bravo, Soon-Yong Kwon, Holm Zaehres, Taehee Lee, Chan Young Park, Hyun-Wook Kang, Hans R. Schöler, Jeong Beom Kim

**Affiliations:** 1 Hans Schöler Stem Cell Research Center (HSSCRC), School of Life Sciences, Ulsan National Institute of Science and Technology (UNIST), 44919 Ulsan, South Korea; 2 SMEs Support Center, Korea Institute of Science and Technology Information, 48058 Busan, South Korea; 3 Group of Computational Biology and Bioinformatics, Biodonostia Health Research Institute, 20014 San Sebastián, Spain; 4 IKERBASQUE, Basque Foundation for Science, Bilbao, Spain; 5 School of Materials Science and Engineering, Ulsan National Institute of Science and Technology (UNIST), 44919 Ulsan, South Korea; 6 Department of Cell and Developmental Biology, Max Planck Institute for Molecular Biomedicine, Röntgenstrasse 20, 48149 Münster, Germany

## Abstract

The maintenance of undifferentiated human pluripotent stem cells (hPSC) under xeno-free condition requires the use of human feeder cells or extracellular matrix (ECM) coating. However, human-derived sources may cause human pathogen contamination by viral or non-viral agents to the patients. Here we demonstrate feeder-free and xeno-free culture system for hPSC expansion using diffusion assisted synthesis-grown nanocrystalline graphene (DAS-NG), a synthetic non-biological nanomaterial which completely rule out the concern of human pathogen contamination. DAS-NG exhibited advanced biocompatibilities including surface nanoroughness, oxygen containing functional groups and hydrophilicity. hPSC cultured on DAS-NG could maintain pluripotency *in vitro* and *in vivo*, and especially cell adhesion-related gene expression profile was comparable to those of cultured on feeders, while hPSC cultured without DAS-NG differentiated spontaneously with high expression of somatic cell-enriched adhesion genes. This feeder-free and xeno-free culture method using DAS-NG will facilitate the generation of clinical-grade hPSC.

Human pluripotent stem cells (hPSC), including human embryonic stem cells (hESC) and human induced pluripotent stem cells (hiPSC), hold great potential for regenerative medicine^1^,^2^. Large-scale hPSC expansion in an undifferentiated state without any pathogen contamination is mandatory for integrating hPSC into the therapeutic applications, which is challenged by current methods. To date, xenogeneic or allogeneic biological substrates are widely used to support hPSC maintenance in an undifferentiated state^1^,^3^,^4^,^5^,^6^,^7^,^8^,^9^. Meanwhile, xenogeneic substrates including mouse embryonic fibroblast (MEF) feeders^1^, or Matrigel and extracellular matrix (ECM) isolated from mouse sarcoma^3^,^5^,^7^ must be avoided for generating clinical-grade hPSC due to the risk of xenogeneic contamination^10^,^11^,^12^. To address this issue, feeder-dependent or feeder-free culture methods under xeno-free condition have been developed by employing allogeneic substrates such as human fibroblast feeder cells^4^,^9^ or purified human ECMs (collagen, fibronectin, laminin or vitronectin)^6^,^8^,^13^ respectively. The allogeneic substrates in a combination with chemically defined xeno-free culture medium can avoid xenogeneic contamination. However, it would be subject to human viral or non-viral contamination to the recipient, which is undesirable for the therapeutic application^12^. In addition, laborious preparation procedure, high manufacture cost and batch-to-batch variability are the drawbacks of allogeneic materials for large-scale hPSC expansion^3^,^6^,^12^. Thus, utilization of biological substrates may not be suitable for generating clinical-grade hPSC.

To exclude the pathogen contamination, synthetic materials have been developed as an alternative for biological substrates due to the advantages in surface modifiability and defined composition^3^,^7^,^14^,^15^. Nevertheless, as some of the synthetic materials require ECM derived synthetic peptides to promote cell adhesion, they are unable to be reused due to the biodegradability of the synthetic peptides^3^, and the cost for synthesizing the peptides is high^3^,^16^. Graphene is a synthetic carbon-based nanomaterial structured in a two-dimensional monolayer sheet of honeycomb lattice with unique mechanochemical properties^17^,^18^. Previous studies have demonstrated differentiation of hESC to cardiomyocytes, and neural stem cells to neurons or oligodendrocytes on graphene layers, thereby demonstrating the biocompatibility of graphene as a stem cell culture substrate^19^,^20^,^21^,^22^. Meanwhile, a number of studies have reported unsuccessful cases of hPSC maintenance on conventional chemical vapor deposition (CVD) graphene without additional ECM coating, possibly due to its intrinsic hydrophobicity^23^,^24^. Critically, ECM-coated CVD graphene may bring pathogen contamination risk. Therefore, we sought to apply our diffusion assisted synthesis (DAS)-grown nanocrystalline graphene (NG) for hPSC culture since DAS-NG possesses topological features suitable for cell adhesion such as intrinsic nanoroughness, oxygen containing functional groups and hydrophilicity^25^. Moreover, DAS method allows to synthesize the NG directly onto the desired substrate at near room temperature at large-scale without a transfer process, thereby simplifying the manufacture procedure^25^.

Here, we successfully established feeder-free and xeno-free culture system for long-term maintenance of hPSC in an undifferentiated state through employing DAS-NG. This is the first report of hPSC maintenance on synthetic graphene surface without ECM coating, which allows the generation of clinical-grade hPSC at large-scale on a pathogen free culture platform.

## Results

### Preparation and characterization of DAS-NG coated culture substrates

**Figure 1:**
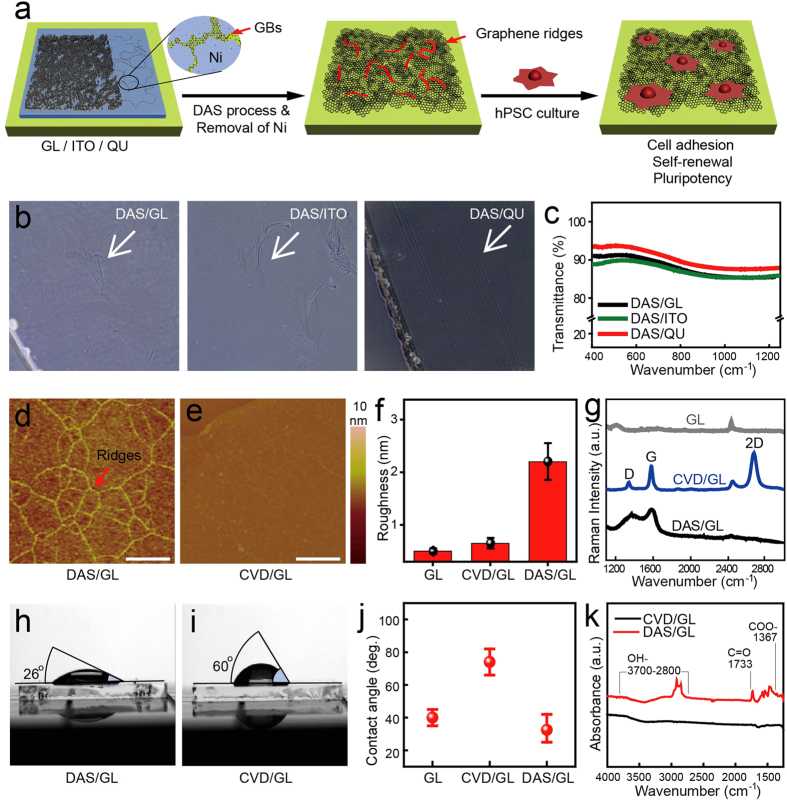
Structural and optical properties of DAS-NG coated culture substrates. (**a**) Schematic diagrams of diffusion assisted synthesis-grown nanocrystalline graphene (DAS-NG) preparation on transparent substrates including GL, ITO, and QU for hPSC cultivation. (GBs, Grain boundaries; Ni, Nickel). (**b**) Optical microscopy images of DAS-NG layers grown at 260 °C on GL, ITO and QU. Graphene layers are indicated with white arrows. (**c**) Transmittances of DAS/GL (black), DAS/ITO (green) and DAS/QU (red). (**d**,**e**) AFM images of (**d**) 3 dimensional DAS-NG layers on GL with high-density multilayer graphene ridges (red arrow) and (**e**) 2 dimensional CVD graphene layers on GL. (**f**) Plot of surface Root-mean-square roughness from AFM images (5 × 5 μm^2^) of GL, CVD/GL and DAS/GL. (**g**) Raman spectra of GL (grey), CVD/GL (blue) and DAS/GL (black). (**h**,**i**) Images of water drop (40 μl on 1.5 × 1.5 cm^2^) contact angle on (**h**) DAS/GL and (**i**) CVD/GL. (**j**) Plot of water contact angle measurements on bare GL, CVD/GL and DAS/GL. (**k**) FT-IR spectra of CVD/GL and DAS/GL. Scale bar, 1 μm (**d**,**e**). Data are presented as mean ± s.e.m (n = 3) (**f**,**j**).

Graphene typically has flat surface and hydrophobic nature, which requires either oxidation process or extracellular matrix (ECM) coating to allow focal adhesion of human pluripotent stem cell (hPSC). To modify the surface morphology and enhance the hydrophilicity of graphene for hPSC cultivation, we employed diffusion-assisted synthesis (DAS) method to grow nanocrystalline graphene (NG). We coated DAS-grown NG (DAS-NG) directly onto glass (GL), indium-tin-oxide (ITO) and quartz (QU) plates at 260 °C for 60 minutes to examine adhesiveness and transparency of DAS-NG on various culture plates (Fig. 1a). Briefly, polycrystalline nickel (Ni) films were deposited onto the plates at room temperature, and the carbon (C) atoms dissociated from graphite powder were diffused through the grain boundaries (GBs) of Ni during the DAS process^25^. Upon reaching the Ni-substrate interface, C atoms precipitate out as graphene at the GBs and growth occurs via lateral diffusion along the interface. Therefore, the resulting films contained uniform naturally-formed multilayer regions at GBs, referred to as graphene ridges, and the density of graphene ridges can be increased by decreasing the average grain size of graphene in the DAS process. We confirmed tightly adhered graphene layers on GL (DAS/GL), ITO (DAS/ITO) and QU (DAS/QU) plates by optical microscopy (Fig. 1b). The resulting DAS-NG layers coated on plates showed ~90.4 ± 4.04% transmittance at 550 nm, suitable for optical imaging (Fig. 1c). We measured surface roughness of DAS-NG to find applicability of DAS-NG for cell culture through atomic force microscopy (AFM) imaging. AFM images revealed three dimensional (3D) surface topography of DAS-NG layers with neighboring multilayered graphene ridges (Fig. 1d and Fig. S1a,b) contrast to monolayer flat surface of chemical vapor diffusion (CVD) graphene (Fig. 1e). However, surface morphology of DAS-NG layer on ITO was hard to distinguish due to intrinsic roughness of underlying ITO plate (Rq ~ 2.6 nm) (Fig. S1a). Root-mean-square roughness (Rq) measured by AFM showed that DAS/GL (2.2 ± 0.35 nm) was 4 times higher than those of CVD coated GL (CVD/GL) (0.65 ± 0.1 nm) and bare GL substrate (0.5 ± 0.05 nm), which indicates the presence of graphene ridges on DAS-NG layers (Fig. 1f and Fig. S1c). All of DAS-NG yielded surface layers of 4.1 ± 1.4 nm and graphene ridges of 8.9 ± 3.2 nm, respectively. Raman structure of DAS-NG layers has the following characteristics; two peaks centered at ~1,350 cm^−1^ (the D band) and at ~1,590 cm^−1^ (the G band) with a relatively large full width at half maximum and an *I*_*D*_*/I*_*G*_ ratio of ~1.0 ± 0.2, which is typical for NG^25^ (Fig. 1g and Fig. S1d). Next, we examined surface hydrophilicity of DAS-NG layers through measuring water contact angles. DAS/GL exhibited a relatively lower contact angle (26 ± 8°) (Fig. 1h,j) than CVD graphene (60 ± 8°) (Fig. 1i,j) or bare GL (40 ± 5°) (Fig. 1j), suggesting the attachment of foreign species onto the surface of the DAS-NG, which enhanced hydrophilicity of DAS-NG. We further investigated the presence of foreign chemical species on the surface of DAS-NG using Fourier transform-infrared (FT-IR) spectroscopy. We constantly found various vibration modes of oxygen-containing functional groups on the surface of DAS-NG including carboxyl group (COO–) at 1,367 cm^−1^, carbonyl group (C = O) at 1,733 cm^−1^ and hydroxyl group (O–H) at 2,800~3,700 cm^−1^ in repeated measurements (n = 3) that were absent on CVD graphene (Fig. 1k). Considering the high affinity of O atoms to C atoms^26^, we inferred that the O atoms in the resulting DAS-NG layers have diffused from the interior of the as-deposited Ni films during the DAS process. On the basis of structural and optical characterizations, we concluded that the DAS-NG layers possess more favorable microenvironment for hPSC adhesion including 3D topography and hydrophilicity than conventional CVD graphene layers.

### Establishment of feeder- and xeno-free culture system for hPSC on DAS-NG

**Figure 2:**
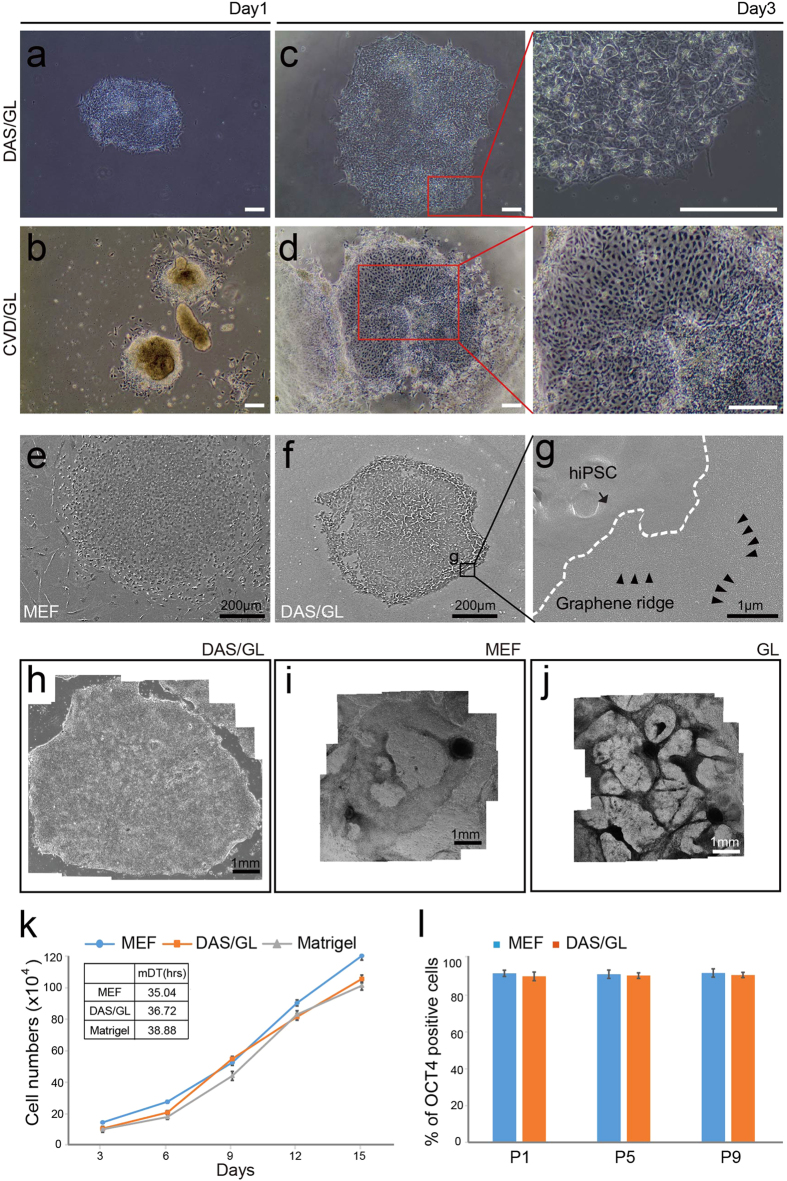
Feeder-free cultivation of hPSC on DAS-NG. (**a**,**b**) Morphology of hiPSC seeded on (**a**) DAS/GL and (**b**) CVD/GL at day 1. (**c**,**d**) High magnification of hiPSC grown on (**c**) DAS/GL and (**d**) CVD/GL at day 3. (**e**–**g**) SEM images of hiPSC cultured on (**e**) MEF, (**f**) DAS/GL at day 3 and (**g**) Zoomed inset shows hiPSC (arrow) attached on graphene ridges (arrow heads) of DAS-NG. (**h**–**j**) Morphology of hiPSC colonies cultured on (**h**) DAS/GL, (**i**) MEF and (**j**) bare GL at 2 weeks. (**k**) The growth rates and mean doubling times (mDT) of hiPSC cultured on MEF, Matrigel or DAS/GL over 15 days. The points refer to the cell number of hiPSC every 3 days. The inset table represents mean doubling time. Data are presented as mean ± s.e.m (n = 3). (**l**) Percentage of OCT4+ hiPSC cultured on MEF or DAS/GL at P1, P5 and P9. Data are presented as mean ± s.e.m (n = 3). Scale bar, 200 μm (**a**–**f**), 1 μm (**g**), 1 mm (**h**–**j**).

To examine the biocompatibility of DAS-NG as a feeder-free culture platform for human pluripotent stem cells (hPSC), we seeded human induced pluripotent stem cells (hiPSC) generated from our previous report^27^ and H9 human embryonic stem cells (hESC) on DAS-NG or CVD graphene-coated substrates without ECM coating in chemically defined xeno-free culture medium supplemented with Knockout serum replacement xeno-free, FGF2, Activin A and TGF-β1. hiPSC showed attachment on all DAS-NG layers within 24 hours without ECM coating (Fig. 2a and Fig. S2a,b), while CVD graphene exhibited poor focal adhesion (Fig. 2b). At day 3, hiPSC colonies grown on DAS-NG showed the typical undifferentiated hPSC morphology with a high nuclear to cytoplasm ratio (Fig. 2c and Fig. S2c,d) similar to those cultured on MEF (Fig. S2e–g). In contrast, hiPSC cultured on CVD graphene underwent spontaneous differentiation (Fig. 2d). The focal adhesion of hiPSC on DAS-NG layer was examined by scanning electron microscopy (SEM). hiPSC exhibited tight adhesion, which is comparable to the attachment of hiPSC cultured on MEF (Fig. 2e–g). We next examined whether the undifferentiated state of hiPSC can be stably maintained for the long-term period (2 weeks) on DAS-NG. hiPSC colonies were expanded into large colonies with typical hPSC morphology on all DAS-NG coated substrates after 2 weeks of cultivation (Fig. 2h and Fig. S3a,b). However, hiPSC co**-**cultured with MEF on GL, ITO and QU substrates were partially differentiated (Fig. 2i and Fig. S3c,d), and hiPSC cultured on all bare substrates without DAS-NG coating underwent spontaneous differentiation (Fig. 2j and Fig. S3e,f). Colony sizes of hiPSC and hESC were measured to analyze the proliferation capacity. The colony sizes were ranged from 3.94 ± 0.11 to 5.45 ± 0.1 mm in diameter on DAS-NG similar to hESC cultured on MEF (Table S1). Importantly, hiPSC could maintain the undifferentiated morphology over multiple passages (>10 passages) after the long-term cultivation (Fig. S3g–l) and multiple freeze-thaw cycles (data not shown). The growth rate of hiPSC cultured on DAS-NG was evaluated every 3 days for 15 days, and we calculated the mean doubling time (mDT) from the growth curve. The mDT of hiPSC cultured on DAS-NG was measured as 36.72 hours and it was comparable with those cultured on MEF (mDT = 35.04 hrs) or Matrigel (mDT = 38.88 hrs) (Fig. 2k). To quantify the number of undifferentiated hiPSC on DAS-NG throughout the passages, we counted OCT4^+^ cells in each colony at passage 1, 5 and 9 (Fig. S3m). The percentage of OCT4^+^ cells on DAS-NG was similar to those cultured on MEF (Fig. 2l). Taken together, the similarity in colony morphology, percentage of OCT4^+^ cells and mDT of hiPSC cultured on DAS-NG in comparison to those cultured on MEF showed that DAS-NG enables the long-term cultivation of hPSC as a feeder-free culture substrates.

### Maintenance of hPSC pluripotency on DAS-NG

**Figure 3:**
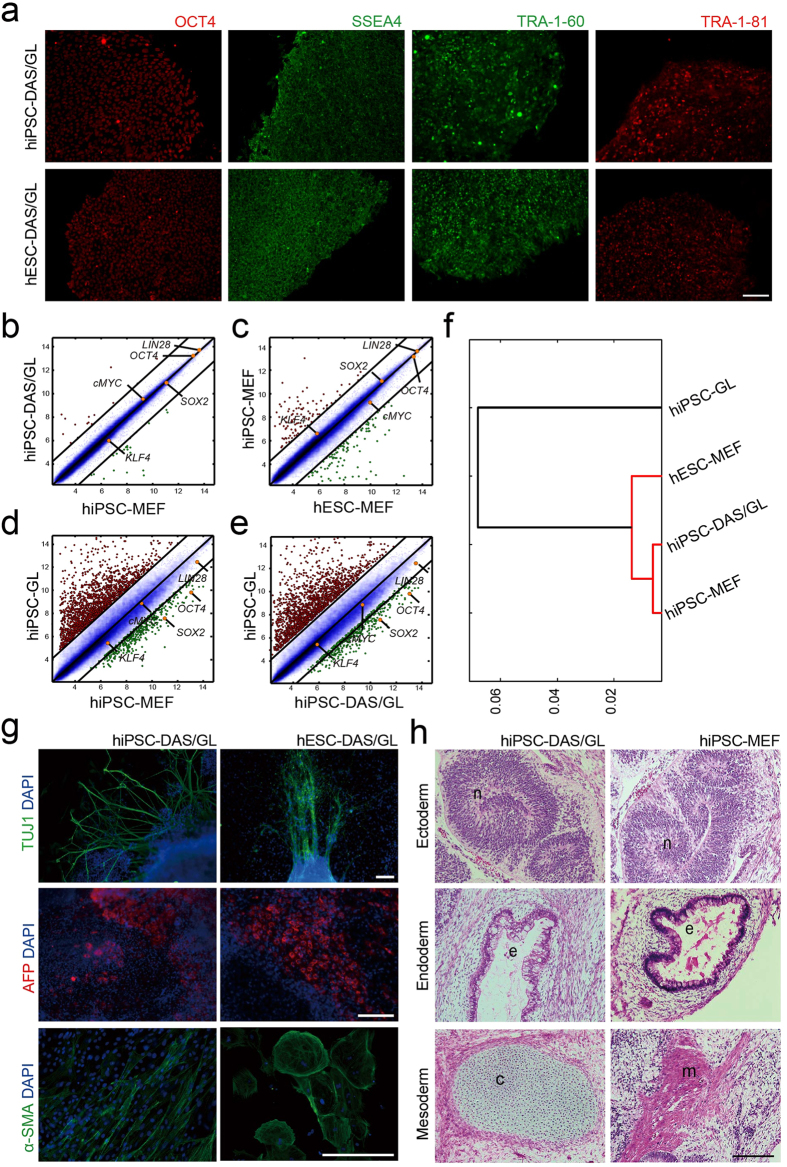
Characterization of hiPSC cultured on DAS/GL. (**a**) Fluorescence images of immunostained hiPSC or hESC with the pluripotency markers (OCT4, SSEA4, TRA-1-60 and TRA-1-81). (**b**–**e**) Pairwise scatter plots comparing global gene expression profile between (**b**) hiPSC-DAS/GL and hiPSC-MEF, (**c**) hiPSC-MEF and hESC-MEF, (**d**) hiPSC-GL and hiPSC-MEF, and (**e**) hiPSC-GL and hiPSC-DAS/GL. Distributions of plutipotency marker genes (*OCT4, SOX2, cMYC, KLF4* and *LIN28*) are indicated in the scatter plots. The black lines indicate the boundaries of two-fold changes in gene expression level. Gene expression levels are shown in log_2_ scale. (**f**) Hierarchical clustering analysis of hiPSC-MEF, hiPSC-DAS/GL, hESC-MEF and hiPSC-GL. (**g**) *In vitro* differentiation analysis of hiPSC-DAS/GL and hESC-DAS/GL stained with three germ layer markers, TUJ1 (ectoderm), AFP (endoderm) and α-SMA (mesoderm) after embryoid body (EB) formation. Cells were counterstained with DAPI. (**h**) Teratoma formation observed at 6 weeks after transplantation of hiPSC-DAS/GL and hiPSC-MEF into SCID mice. Shown is a haematoxylin and eosin stained teratoma sections containing all three germ layers; ectoderm (neural rosette, n), endoderm (respiratory epithelium, e) and mesoderm (cartilage, c; skeletal muscle, m). Scale bar, 150 μm (**a**), 100 μm (**g**,**h**).

We examined cellular and molecular properties of hiPSC cultured on DAS-NG after 2 weeks of cultivation. Remarkably, hPSC retained expression of the pluripotency markers (OCT4, SSEA-4, TRA-1-60 and TRA-1-81) on DAS-NG (Fig. 3a). We further characterized *in vitro* and *in vivo* pluripotency of hPSC cultured on DAS-NG. hiPSC cultured on all three DAS-NG coated substrates (hiPSC-DAS/GL, hiPSC-DAS/ITO and hiPSC-DAS/QU) could maintain the expression of the pluripotency marker genes including *OCT4, NANOG, SOX2* and *LIN28* in a similar level to hiPSC cultured on MEF (hiPSC-MEF), while hiPSC cultured on bare glass (hiPSC-GL) exhibited down-regulation of these genes (Fig. S4a). To analyze the molecular characteristics of hiPSC-DAS/GL, we compared the global gene expression patterns between hiPSC-DAS/GL, hESC-MEF, hiPSC-MEF and hiPSC-GL. Pairwise scatter plots showed a high similarity of global gene expression pattern between hiPSC-DAS/GL and hiPSC-MEF (Fig. 3b), which is in the similar range of hiPSC-MEF and hESC-MEF (Fig. 3c). In contrast, hiPSC-GL showed a low similarity with hiPSC-MEF or hiPSC-DAS/GL (Fig. 3d,e). Consistent with the pairwise scatter plot results, the hierarchical clustering analysis also showed tight clustering of hiPSC-DAS/GL with the wild-type hPSC (hiPSC-MEF and hESC-MEF) which was distinct from hiPSC-GL (Fig. 3f). Moreover, we generated a heat map with 22 pluripotent stem cell-enriched genes and 19 somatic cell-enriched genes (Fig. S4b). Wild-type hPSC and hiPSC-DAS/GL shared high expression in pluripotent stem cell-enriched genes and low expression in somatic cell-enriched genes, which is distinct from the expression pattern of hiPSC-GL. Next, we evaluated the differentiation potential of hiPSC-DAS/GL and hESC-DAS/GL into all three germ layers *in vitro* by hanging drop method. After 2 weeks of *in vitro* differentiation, we confirmed the expression of all three germ layer markers, TUJ1 (ectoderm), AFP (endoderm) and α-SMA (mesoderm) by immunostaining (Fig. 3g). Also, the differentiation potential of hiPSC-DAS/GL *in vivo* was evaluated via teratoma formation assay. hiPSC-DAS/GL or hiPSC-MEF were injected subcutaneously into severe combined immunodeficient (SCID) mice to investigate the differentiation potential *in vivo*. After 6 weeks of injection, we observed formation of teratomas that contain tissue structures of neural rosette (ectoderm), respiratory epithelium (endoderm) and muscle (mesoderm) representing all three germ layers in hiPSC-DAS/GL injected mice as in hiPSC-MEF injected mice (Fig. 3h). These results clearly show that pluripotency of hPSC can be stably maintained in feeder-free culture condition using DAS-NG.

### Expression of hPSC-enriched focal adhesion gene on DAS-NG

**Figure 4:**
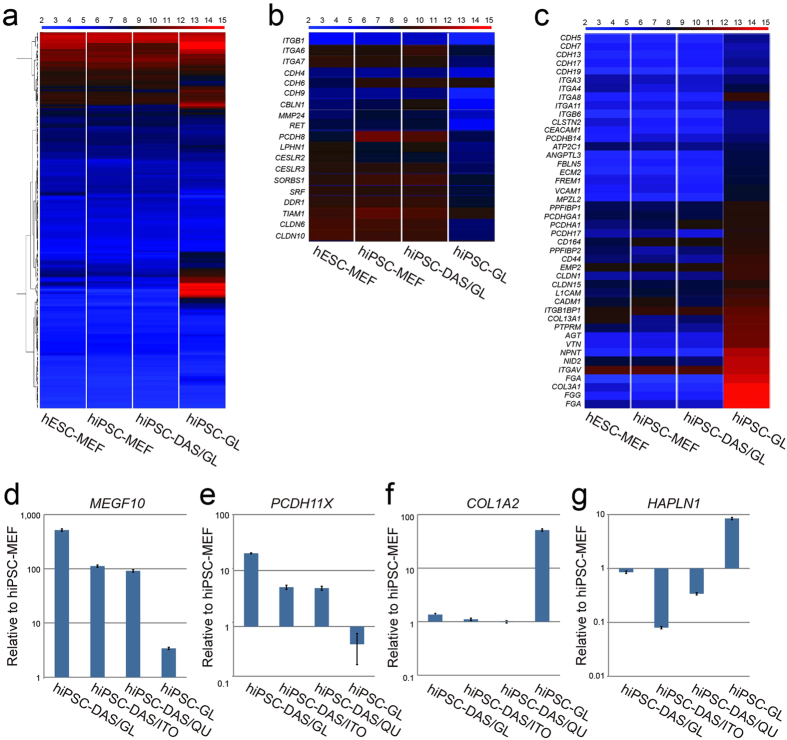
hPSC cell adhesion gene expression on DAS-NG. (**a**–**c**) Heat maps of (**a**) 254 cell adhesion-related genes, (**b**) selected 19 hPSC-enriched cell adhesion genes and (**c**) 44 somatic-enriched cell adhesion genes within the hESC-MEF, hiPSC-MEF, hiPSC-DAS/GL and hiPSC-GL. A color bar (top) indicates the color code gene expression in log_2_ scale. (**d**–**g**) qRT–PCR analysis for hPSC-enriched cell adhesion genes (**d**) *MEGF10* and (**e**) *PCDH11X*, and somatic-enriched cell adhesion genes (**f**) *COL1A2* and (**g**) *HAPLN1* in hiPSC-DAS/GL, hiPSC-DAS/ITO, hiPSC-DAS/QU and hiPSC-GL relative to hiPSC-MEF. Transcript levels are normalized to *GAPDH* and represented in the logarithmic scale. Data are presented as mean ± s.e.m (n = 3) (**d**–**g**).

We investigated how DAS-NG can support the maintenance of pluripotency by analyzing the cell adhesion-related gene expression since cell adhesion molecules such as integrin families play important role in regulation of hPSC focal adhesion and self-renewal^14^,^28^. We analyzed the expression profile of total 254 genes involved in the cell adhesion including 92 cell-matrix adhesion genes (GO:0007160) and 162 cell-cell adhesion genes (GO:0098609) by microarray analysis. We compared the cell adhesion-related gene expression patterns in hiPSC cultured on DAS-NG to those cultured on MEF and spontaneously differentiated hiPSC cultured on bare GL. The heat map highlighted clusters of the genes that showed very similar patterns among hiPSC-DAS/GL and wild-type hPSC, but distinct from hiPSC-GL (Fig. 4a). Also, the clusters of genes that showed high expression only in the spontaneously differentiated hiPSC-GL were observed (Fig. 4a). The cell type specific expression of the cell adhesion-related genes in our list was identified from analyzing the previously reported GEO database (GSE23034)^29^. From the 254 genes, we selected 19 hPSC-enriched cell adhesion genes that are highly expressed in hiPSC-DAS/GL and wild type hPSC (Fig. 4b and Table S2), which is distinct from hiPSC-GL (*P*-value < 0.05), and also 44 somatic-enriched cell adhesion genes highly expressed only in differentiated hiPSC-GL (*P*-value < 0.05) (Fig. 4c and Table S3)^29^. Interestingly, from the 19 selected gene, integrin α_6_ and β_1_, which is known to have an important role in cell adhesion of hPSC to laminin and the regulation of hESC self-renewal^30^,^31^ showed a similar expression level in hiPSC-DAS/GL with hPSC-MEF (Fig. 4b and Table S2). We also measured and compared mRNA expression level of hPSC-enriched cell adhesion genes reported in other studies^32^,^33^ by qRT-PCR analysis. We identified hPSC-enriched cell adhesion genes, *MEGF10* and *PCDH11X*^29^,^32^,^33^ were highly expressed in hiPSC cultured on all DAS-NG coated substrates (Fig. 4d,e). Meanwhile, the expression level of somatic-enriched cell adhesion genes, *COL1A2* and *HAPLN2*^29^,^34^ were up-regulated in hiPSC-GL while down-regulated in hiPSC grown on all DAS-NG coated substrates (Fig. 4f,g). Hence, these results show that DAS-NG provides suitable surface topography to maintain expression of hPSC-enriched cell adhesion genes and it may contribute to maintenance of hiPSC in an undifferentiated state.

## Discussion

Here we developed a new feeder-free culture platform for hPSC cultivation by employing synthetic DAS-NG in combination with chemically defined xeno-free culture medium without biological ECM coating. DAS-NG successfully supported the focal adhesion and the long-term cultivation of hPSC in an undifferentiated state. In comparison to the conventional feeder-dependent culture method, DAS-NG showed superior in maintenance of the typical undifferentiated hPSC morphology for long-term culture. Moreover, the growth rate and mean doubling time of hiPSC cultured on DAS-NG were comparable with those cultured on MEF or Matrigel (Fig. 2k), which indicate that DAS-NG can maintain consistent metabolic rate of hiPSC.

hiPSC cultured on DAS-NG showed similar expression pattern of cell adhesion-related genes with those cultured on MEF. Especially, the expression of integrin α6 and β1, which are known to facilitate the adhesion of hPSC to laminin, thereby supporting maintenance of pluripotency^30^,^35^, was high (Fig. 4b). Thus, we suppose that DAS-NG possesses the physiochemical features comparable with laminin for the adhesion of hPSC.

Synthemax is one of the synthetic materials for hPSC culture in feeder-free conditon^36^, however, the cost for production is expensive^3^,^16^ and the synthetic peptides on Synthemax can be degraded by metallopeptidase that is secreted from the cultured cells^3^,^16^,^37^. In contrast, synthesis of graphene is cost-effective and simple^25^,^38^ which enables the large-scale production of graphene layers for hPSC culture. Also, the surface properties of graphene are persisted during the long-term hPSC culture due to the non-biodegradability^39^ which can allow low variation to maintain the pluripotency as a reliable condition. In addition, graphene has unique chemical and physical properties that regulate the differentiation of hPSC^39^. For instance, graphene surface is modifiable by proteins that are used to differentiate hPSC such as BMP-2 or heparin due to noncovalent interactions^39^,^40^,^41^,^42^,^43^. Moreover, graphene is an electrical conductor^39^ that can be used to measure the cellular electrical activities^22^,^44^ or to give electrical stimulation to the hPSC which can enhance the differentiation of hPSC to neurons or cardiomyocytes^45^,^46^,^47^.

Several studies have reported that the nanoroughness and hydrophilicity of graphene are correlated with the hPSC focal adhesion which is associated with pluripotency^48^,^49^,^50^. We found that graphene ridges enhanced the nanoroughness (Fig. 1d), and the naturally obtained oxygen containing functional groups increased hydrophilicity of DAS-NG during graphene diffusion process (Fig. 1k). Meanwhile, graphene oxide (GO) is also hydrophilic^43^, nanoroughness is lower than DAS-NG^50^. The topology of DAS-NG may contribute to maintain pluripotency of hPSC while GO promotes the differentiation toward the ectoderm lineage^43^. However, how the topological difference of graphene regulates pluripotency remains to be elucidated.

Previous studies demonstrated CVD graphene requires biological ECM coating to allow focal adhesion of hPSC^19^,^23^. In particular, Lee *et al.* reported hESC cultured on uncoated CVD graphene were not maintained within two days of culture^19^. In contrast, DAS-NG was sufficient to support the adhesion and long-term culture of hPSC without ECM coating (Fig. 2a,h).

In summary, DAS-NG as feeder-free culture substrate for hPSC is a synthetic material freed from pathogen contamination that has biocompatibilities including nanoroughness, hydrophilicity and oxygen containing functional groups, and is simple and cost-effective in manufacturing process^3^,^25^, thereby allowing large-scale culture of hPSC in clinical grade.

## Methods

### Preparation of DAS-NG coated culture templates

For DAS-NG preparation, 50-nm-thick film of polycrystalline Ni (poly-Ni) was deposited via electron-beam evaporation at room temperature on Glass (GL), indium-tin-oxide (ITO) and Quartz (QU) substrates. The Ni surface was coated with the graphite powder (SIGMA), and then the graphite powder was pressed onto the Ni/substrates assembly. At temperatures below 260 ^o^C, C atoms in the samples began to diffuse through the Ni along the GBs. As the diffusing C atoms reached on Ni/substrates interface, they created a thin film of NG at the Ni/substrates interface. Pressure of ~1 MPa was uniformly applied by mechanically clamping the C-Ni/plate diffusion couple using a molybdenum holding stage. The pressure promotes the diffusion of C through the Ni film. Following NG growth, the samples were cleaned via sonication in deionized water, and the Ni films were removed by etching in an aqueous solution of FeCl_3_, leaving behind a NG film on desired substrates.

### Preparation of CVD-graphene coated culture templates

Conventional graphene layers were grown on a 80-nm-thick Pt (111) film on SiO_2_/Si (GMEK Incorporation) using a low-pressure CVD system. After the Pt substrates were loaded into a quartz tube in LP-CVD, the samples were heated to the process temperature of 975 °C and maintained for 10 min under CH_4_/H_2_ gas mixture (5 and 50 sccm, respectively) to form graphene. Following the graphene growth, the quartz tube was cooled down to room temperature. Poly methyl methacrylate (PMMA) was used to transfer graphene onto the transparent (GL, ITO, QU) substrates. A layer of PMMA was spin-coated onto the graphene/Pt films to act as a supporting layer. A thermal-assisted transfer method was then used to separate between PMMA/graphene and the Pt film, after which the PMMA/graphene stack was transferred to the transparent substrates^51^. Finally, the PMMA was removed using acetone, leaving behind the graphene film on the transparent substrates.

### DAS/CVD-graphene characterization

DAS-NG and transferred CVD graphene layers on the transparent substrates were analyzed by AFM (Veeco Multimode V) to observe surface morphology and to measure surface roughness. The AFM was operated using tapping mode to acquire a scan size of 5 × 5 μm^2^. The presence of graphene frameworks were confirmed by Raman spectroscopy. The Raman spectroscopy was carried out on a WiTec alpha 300R M-Raman system with a 532 nm excitation wavelength (2.33 eV). A laser spot had a dimension of ~640 nm for a ×50 objective lens with numerical aperture of 0.5 and the laser power was ~2 mW. To investigate the presence and chemical states of foreign species in the graphene framework, the Fourier transform infrared (FT-IR) spectra of the samples were measured using the Agilent Cary 670-IR, vacuum FT-IR spectrometer over a range from 650 to 4000 cm^−1^. The water drop contact angles were observed by a KRUSS DSA-100 (Germany) drop shape measurement. The optical transmittances of the samples were measured using UV–vis spectroscopy (Varian, Cary 5000 model) between 200 and 800 nm in dual-beam mode.

### hPSC cultivation

The generation and characterization of hiPSC from human neural stem cell (NSC) with *OCT4* and *KLF4* (NSC-2F-iPSC) or only *OCT4* (NSC-1F-iPSC) by our group has been published in elsewhere^27^. NSC-2F-iPSC (hiPSC) and H9 human ESC (WiCell) were seeded onto DAS-NG coated substrates (Glass, ITO, and Quartz), or CVD-graphene coated glass, or mitomycin-C treated CF1 mouse feeders. Human pluripotent stem cells were cultured in knockout DMEM (GIBCO) supplemented with 20% KnockOut serum replacement xeno-free (GIBCO), 1 mM L-glutamine, 1% penicillin/streptomycin (GIBCO), 1% MEM-non essential amino acid (GIBCO), 0.1 mM β-mercaptoethanol (Sigma-Aldrich), 5 ng/ml human basic fibroblast growth factor (Peprotech), 100 ng/ml Activin A (Peprotech) and 2 ng/ml TGF-β1 (Peprotech). Only undifferentiated colonies were subcultured by hand-picked mechanical method for >10 passages and cryopreserved. Cells at 2 weeks of culture were used for characterization.

### Growth curve and mean doubling time

Cell growth rate and mean doubling time were analyzed every 3 days for 15 days. hiPSC colonies cultured on MEF, DAS-NG or Matrigel (BD Biosciences) were disassociated into single cells using 0.05% trypsin/EDTA, and the cells were manually counted using a hemocytometer (Marienfeld). The average cell numbers at each passage (n = 3) were plotted. The mean doubling times were calculated from the plotted growth curve.

### Quantitative real time PCR

DNA-free total RNA was extracted using the RNeasy mini kit (Qiagen). We performed cDNAs synthesis using SuperScript^®^ III reverse transcriptase (Invitrogen) with 500 ng of total RNA per reaction. Synthesized cDNA was purified using PCRquick-spin (iNtRON) columns and purified cDNA (25 ng) was used as a template in the LightCycler 480 SYBR Green I Master (Roche). Experiments were performed in triplicates and were normalized to the housekeeping gene *GAPDH*. Gene expressions were measured by Ct calculating method. The primer sequences for each gene in the present study are listed in Table S4.

### Immunocytochemistry staining

Human pluripotent stem cells were fixed for 10 minutes in 4% paraformaldehyde (PFA) and permeabilized with 0.1% Triton X-100 for 10 minutes. The cells were incubated in 4% FBS blocking solution for 30 minutes and incubated in primary antibodies for 1 hour at room temperature. The primary antibody were specific for OCT3/4 (Santa Cruz, 1:200), SSEA-4 (Millipore, 1:200), TRA-1-60 (Millipore, 1:200), TRA-1-81 (Millipore, 1:200), TUJ1 (Millipore, 1:200), a-Smooth Muscle actin (Abcam, 1:250), AFP (DAKO, 1:200). The secondary antibodies were diluted in PBS and stained for 1 hour at the following concentration: Alexa Fluor 488/568 anti-mouse IgG1, IgG2a, IgG3, IgM, anti-goat IgG, anti-rabbit IgG (Invitrogen, 1:1000). The primary antibodies used in the present study are listed in Supplementary Table S5. The nuclei were counterstained with DAPI (Invitrogen) and observed under fluorescence microscope. At the end of Passage 1, 5 and 9 the number of OCT4^+^ cells and cell nuclei (DAPI) in each colony cultured on DAS-GL (n = 3) were counted using ImageJ software. The percentage of OCT4^+^ cells in each colony was calculated to quantify the number of hPSC in each passage.

### *In vitro* differentiation

Embryonic body (EB) formation was initiated by harvesting cells and transfer to human embryonic stem cell medium without human basic-FGF using the hanging-drop method by placing 20ul drop containing cells on the lid of culture plate. After 1 week of culture the mass of cells was transferred into gelatin-coated plates and cultured until the EBs adhere to plate tightly.

### Teratoma formation

All mice were housed on a 12 hr light/dark cycle with free access to water and food. The experimental procedures were carried out in accordance with the approved guidelines and all protocols were approved by the Animal Care and Use Committee of Ulsan National Institute of Science and Technology (Ulsan, South Korea). hPSCs maintained on DAS-NG or MEF for 14 days (3–5 × 10^6^ cells/mouse) were injected into the subcutaneous of dorsal flank of severe combined immunodeficient (SCID) mice. Teratoma were harvested after 6–8 weeks of injection and fixed in 4% PFA overnight before embedding in paraffin. Paraffin sections were stained with haematoxylin and eosin.

### Microarray data processing

The normalization was calculated with the RMA (Robust Multi-array Analysis) algorithm^52^. Data post-processing and graphics was performed with in-house developed functions in Matlab. Hierarchical clustering of genes and samples was performed with one minus correlation metric and the unweighted average distance (UPGMA) (also known as group average) linkage method. Total RNA was isolated using RNeasy mini kit (Qiagen) following the manufacturer’s instruction. Samples were hybridized to Affymetrix Human genome U133 plus 2.0 chip. The normalization calculated with Robust Multi-array Analysis (RMA) algorithm^52^.

### Statistical analysis

All experiments were repeated three times and data were analyzed via student’s *t-*tests. The results are expressed as mean values. *P*-value < 0.05 was considered significant.

## Additional Information

**Accession codes:** The microarray data reported in this article have been deposited in the NCBI Gene Expression Omnibus database under accession number GSE66439.

**How to cite this article**: Lee, H. *et al.* Establishment of feeder-free culture system for human induced pluripotent stem cells on DAS nanocrystalline graphene. *Sci. Rep.* **6**, 20708; doi: 10.1038/srep20708 (2016).

## Supplementary Material

Supplementary Information

## References

[b1] Thomson J. A. *et al.* Embryonic stem cell lines derived from human blastocysts. Science (New York, N.Y.) 282, 1145–1147 (1998). 10.1126/science.282.5391.1145 9804556

[b2] Wu S. M. & Hochedlinger K. Harnessing the potential of induced pluripotent stem cells for regenerative medicine. Nature cell biology 13, 497–505 (2011). 21540845 10.1038/ncb0511-497 PMC3617981

[b3] Villa-Diaz L. G., Ross A. M., Lahann J. & Krebsbach P. H. Concise review: The evolution of human pluripotent stem cell culture: from feeder cells to synthetic coatings. Stem cells (Dayton, Ohio) 31, 1–7 (2013). 10.1002/stem.1260 PMC3537180 23081828

[b4] Richards M., Fong C. Y., Chan W. K., Wong P. C. & Bongso A. Human feeders support prolonged undifferentiated growth of human inner cell masses and embryonic stem cells. Nature biotechnology 20, 933–936 (2002). 10.1038/nbt726 12161760

[b5] Xu C. *et al.* Feeder-free growth of undifferentiated human embryonic stem cells. Nature biotechnology 19, 971–974 (2001). 10.1038/nbt1001-971 11581665

[b6] Nakagawa M. *et al.* A novel efficient feeder-free culture system for the derivation of human induced pluripotent stem cells. Scientific Reports 4, 3594 (2014). 24399248 10.1038/srep03594 PMC3884228

[b7] Zonca M. R. Jr & Xie Y. Chemically modified micro-and nanostructured systems for pluripotent stem cell culture. BioNanoScience 2, 287–304 (2012).

[b8] Amit M. & Itskovitz-Eldor J. Feeder-free culture of human embryonic stem cells. Methods in enzymology 420, 37–49 (2006). 17161692 10.1016/S0076-6879(06)20003-X

[b9] Takahashi K., Narita M., Yokura M., Ichisaka T. & Yamanaka S. Human induced pluripotent stem cells on autologous feeders. PloS one 4, e8067 (2009). 19956543 10.1371/journal.pone.0008067 PMC2780725

[b10] Martin M. J., Muotri A., Gage F. & Varki A. Human embryonic stem cells express an immunogenic nonhuman sialic acid. Nature medicine 11, 228–232 (2005). 10.1038/nm1181 15685172

[b11] Rottem S. & Barile M. F. Beware of mycoplasmas. Trends in biotechnology 11, 143–151 (1993). 7763647 10.1016/0167-7799(93)90089-R

[b12] Stacey G. N. *et al.* The development of ‘feeder’ cells for the preparation of clinical grade hES cell lines: challenges and solutions. Journal of biotechnology 125, 583–588 (2006). 16690155 10.1016/j.jbiotec.2006.03.011

[b13] Hakala H. *et al.* Comparison of biomaterials and extracellular matrices as a culture platform for multiple, independently derived human embryonic stem cell lines. Tissue engineering. Part A 15, 1775–1785 (2009). 19132919 10.1089/ten.tea.2008.0316

[b14] Lambshead J., Meagher L., O’Brien C. & Laslett A. Defining synthetic surfaces for human pluripotent stem cell culture. Cell Regen 2, 1–17 (2013). 10.1186/2045-9769-2-7 PMC4230363 25408879

[b15] Brafman D. A. *et al.* Long-term human pluripotent stem cell self-renewal on synthetic polymer surfaces. Biomaterials 31, 9135–9144 (2010). 20817292 10.1016/j.biomaterials.2010.08.007 PMC2949524

[b16] Ross A. M., Nandivada H., Ryan A. L. & Lahann J. Synthetic substrates for long-term stem cell culture. Polymer 53, 2533–2539 (2012).

[b17] Rao C. N., Sood A. K., Subrahmanyam K. S. & Govindaraj A. Graphene: the new two-dimensional nanomaterial. Angew Chem Int Ed Engl 48, 7752–7777 (2009). 19784976 10.1002/anie.200901678

[b18] Novoselov K. S. *et al.* Electric field effect in atomically thin carbon films. Science (New York, N.Y.) 306, 666–669 (2004). 10.1126/science.1102896 15499015

[b19] Lee T. J. *et al.* Graphene enhances the cardiomyogenic differentiation of human embryonic stem cells. Biochemical and biophysical research communications 452, 174–180 (2014). 25152405 10.1016/j.bbrc.2014.08.062

[b20] Tang M. *et al.* Enhancement of electrical signaling in neural networks on graphene films. Biomaterials 34, 6402–6411 (2013). 23755830 10.1016/j.biomaterials.2013.05.024

[b21] Shah S. *et al.* Guiding stem cell differentiation into oligodendrocytes using graphene-nanofiber hybrid scaffolds. Advanced materials (Deerfield Beach, Fla.) 26, 3673–3680 (2014). 10.1002/adma.201400523 PMC4048813 24668911

[b22] Park S. Y. *et al.* Enhanced Differentiation of Human Neural Stem Cells into Neurons on Graphene. Advanced Materials 23, H263–H267 (2011). 21823178 10.1002/adma.201101503

[b23] Sebaa M., Nguyen T. Y., Paul R. K., Mulchandani A. & Liu H. Graphene and carbon nanotube–graphene hybrid nanomaterials for human embryonic stem cell culture. Materials Letters 92, 122–125 (2013).

[b24] Sanchez V. C., Jachak A., Hurt R. H. & Kane A. B. Biological interactions of graphene-family nanomaterials: an interdisciplinary review. Chemical research in toxicology 25, 15–34 (2011). 21954945 10.1021/tx200339h PMC3259226

[b25] Kwak J. *et al.* Near room-temperature synthesis of transfer-free graphene films. Nat Commun 3, 645 (2012). 22273683 10.1038/ncomms1650

[b26] Chu J. H. *et al.* Monolithic graphene oxide sheets with controllable composition. Nat Commun 5, 3383 (2014). 24577159 10.1038/ncomms4383

[b27] Kim J. B. *et al.* Direct reprogramming of human neural stem cells by OCT4. Nature 461, 649–643 (2009). 19718018 10.1038/nature08436

[b28] Lee S. T. *et al.* Engineering integrin signaling for promoting embryonic stem cell self-renewal in a precisely defined niche. Biomaterials 31, 1219–1226 (2010). 19926127 10.1016/j.biomaterials.2009.10.054

[b29] Ohi Y. *et al.* Incomplete DNA methylation underlies a transcriptional memory of somatic cells in human iPS cells. Nature cell biology 13, 541–549 (2011). 21499256 10.1038/ncb2239 PMC3987913

[b30] Braam S. R. *et al.* Recombinant vitronectin is a functionally defined substrate that supports human embryonic stem cell self-renewal via alphavbeta5 integrin. Stem cells (Dayton, Ohio) 26, 2257–2265 (2008). 10.1634/stemcells.2008-0291 18599809

[b31] Li L., Bennett S. A. & Wang L. Role of E-cadherin and other cell adhesion molecules in survival and differentiation of human pluripotent stem cells. Cell adhesion & migration 6, 59–70 (2012). 22647941 10.4161/cam.19583 PMC3364139

[b32] Hasegawa Y. *et al.* CCL2 enhances pluripotency of human induced pluripotent stem cells by activating hypoxia related genes. Scientific Reports 4, 5228 (2014). 24957798 10.1038/srep05228 PMC4067614

[b33] Boheler K. R. *et al.* A human pluripotent stem cell surface N-glycoproteome resource reveals markers, extracellular epitopes, and drug targets. Stem cell reports 3, 185–203 (2014). 25068131 10.1016/j.stemcr.2014.05.002 PMC4110789

[b34] Jagtap S. *et al.* Cytosine arabinoside induces ectoderm and inhibits mesoderm expression in human embryonic stem cells during multilineage differentiation. British journal of pharmacology 162, 1743–1756 (2011). 21198554 10.1111/j.1476-5381.2010.01197.x PMC3081118

[b35] Shen H., Zhang L., Liu M. & Zhang Z. Biomedical applications of graphene. Theranostics 2, 283–294 (2012). 22448195 10.7150/thno.3642 PMC3311234

[b36] Melkoumian Z. *et al.* Synthetic peptide-acrylate surfaces for long-term self-renewal and cardiomyocyte differentiation of human embryonic stem cells. Nature biotechnology 28, 606–610 (2010). 10.1038/nbt.1629 20512120

[b37] Fonseca K. B., Bidarra S. J., Oliveira M. J., Granja P. L. & Barrias C. C. Molecularly designed alginate hydrogels susceptible to local proteolysis as three-dimensional cellular microenvironments. Acta biomaterialia 7, 1674–1682 (2011). 21193068 10.1016/j.actbio.2010.12.029

[b38] Baig N. & Kawde A.-N. A novel, fast and cost effective graphene-modified graphite pencil electrode for trace quantification of l-tyrosine. Analytical Methods 7, 9535–9541 (2015).

[b39] Ryu S. & Kim B.-S. Culture of neural cells and stem cells on graphene. Tissue Eng Regen Med 10, 39–46 (2013).

[b40] Utesch T., Daminelli G. & Mroginski M. A. Molecular dynamics simulations of the adsorption of bone morphogenetic protein-2 on surfaces with medical relevance. Langmuir : the ACS journal of surfaces and colloids 27, 13144–13153 (2011). 21958113 10.1021/la202489w

[b41] Lee da Y., Khatun Z., Lee J. H., Lee Y. K. & In I. Blood compatible graphene/heparin conjugate through noncovalent chemistry. Biomacromolecules 12, 336–341 (2011). 21218769 10.1021/bm101031a

[b42] Wheeler S. E. Understanding substituent effects in noncovalent interactions involving aromatic rings. Accounts of chemical research 46, 1029–1038 (2013). 22725832 10.1021/ar300109n

[b43] Lee W. C. *et al.* Origin of enhanced stem cell growth and differentiation on graphene and graphene oxide. ACS nano 5, 7334–7341 (2011). 21793541 10.1021/nn202190c

[b44] Li N. *et al.* Three-dimensional graphene foam as a biocompatible and conductive scaffold for neural stem cells. Scientific Reports 3, 1604 (2013). 23549373 10.1038/srep01604 PMC3615386

[b45] Yamada M. *et al.* Electrical stimulation modulates fate determination of differentiating embryonic stem cells. Stem cells (Dayton, Ohio) 25, 562–570 (2007). 10.1634/stemcells.2006-0011 17110622

[b46] Serena E. *et al.* Electrical stimulation of human embryonic stem cells: cardiac differentiation and the generation of reactive oxygen species. Experimental cell research 315, 3611–3619 (2009). 19720058 10.1016/j.yexcr.2009.08.015 PMC2787733

[b47] Chan Y. C. *et al.* Electrical stimulation promotes maturation of cardiomyocytes derived from human embryonic stem cells. Journal of cardiovascular translational research 6, 989–999 (2013). 24081385 10.1007/s12265-013-9510-z

[b48] Akasaka T., Yokoyama A., Matsuoka M., Hashimoto T. & Watari F. Maintenance of hemiround colonies and undifferentiated state of mouse induced pluripotent stem cells on carbon nanotube-coated dishes. Carbon 49, 2287–2299 (2011).

[b49] Chung C. *et al.* Biomedical applications of graphene and graphene oxide. Accounts of chemical research 46, 2211–2224 (2013). 23480658 10.1021/ar300159f

[b50] Chen G. Y., Pang D. W., Hwang S. M., Tuan H. Y. & Hu Y. C. A graphene-based platform for induced pluripotent stem cells culture and differentiation. Biomaterials 33, 418–427 (2012). 22014460 10.1016/j.biomaterials.2011.09.071

[b51] Choi J.-K. *et al.* Growth of Wrinkle-Free Graphene on Texture-Controlled Platinum Films and Thermal-Assisted Transfer of Large-Scale Patterned Graphene. ACS Nano 9, 679–686 (2015). 25494828 10.1021/nn5060909

[b52] Irizarry R. A. *et al.* Exploration, normalization, and summaries of high density oligonucleotide array probe level data. Biostatistics (Oxford, England) 4, 249–264 (2003). 10.1093/biostatistics/4.2.249 12925520

