# Supplementary material for: Establishment of feeder-free culture system for human induced pluripotent stem cell on DAS nanocrystalline graphene
Source: Sci Rep. 2016 Feb 5;6:20708. doi: 10.1038/srep20708 (PMC4742916; doi:10.1038/srep20708)
Supplement: Supplementary Information [file srep20708-s1.pdf]

Supplementary Information

Establishment of feeder-free culture system for human induced pluripotent stem cells on DAS nanocrystalline graphene

Hyunah Lee, Donggyu Nam, Jae-Kyung Choi, Marcos J. Araúzo-Bravo, Soon-Yong Kwon, Holm Zaehres, Tahee Lee, Chan Young Park, Hyun-Wook Kang, Hans R. Schöler & Jeong Beom Kim

Contents

Supporting Information Figures

Figure S1. Structural and optical properties of DAS/ITO or DAS/QU.................................S1
Figure S2. Morphology of hPSC on DAS/ITO, DAS/QU or MEF..S2
Figure S3. Long-term maintenance of undifferentiated hiPSC on DAS/ITO or DAS/QU….S3
Figure S4. Molecular characterization of hiPSC cultured on DAS/NG……………………..S4

Supporting Information Tables

Table S1. Average colony size of hiPSC and hESC on DAS-NG at day 14...........................S5
Table S2. hPSC enriched cell adhesion gene expression of hiPSC or hESC grown on MEF, DAS/GL or bare GL..S6
Table S3. Three-germ layer enriched cell adhesion gene expression of hiPSC or hESC grown on MEF, DAS/GL or bare GL..S7
Table S4. Quantitative real time PCR primers for cell-adhesion related genes......................S8
Table S5. Primary antibody list..S9

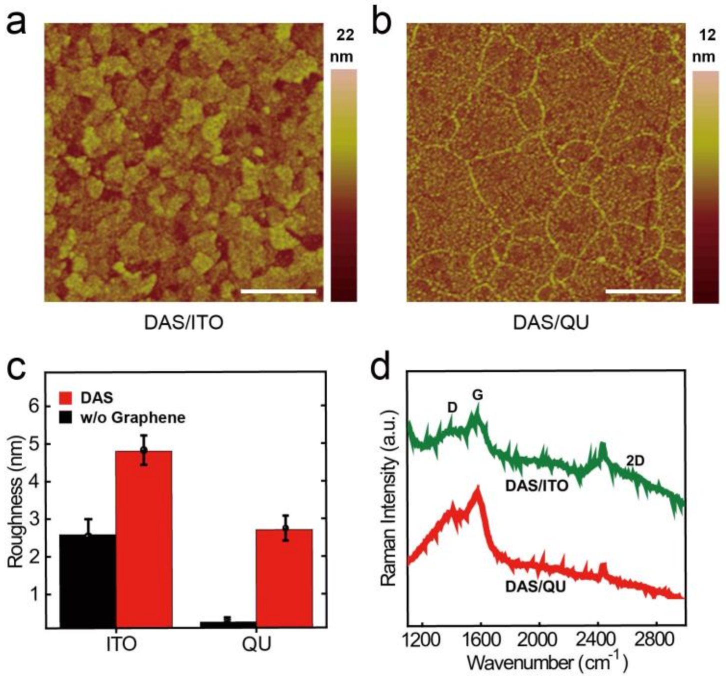

Figure S1. Structural and optical properties of DAS-NG coated ITO or Quartz substrates. (**a**,**b**) AFM images of the NG film coated by the DAS process at T = 260 ^{o}C for 60 min on (**a**) ITO and (**b**) QU plates. Shown is the presence of high-density multilayer graphene ridges. (**c**) Plot of surface RMS roughness from AFM images (5 × 5 μm^2) of bare ITO, DAS/ITO and bare QU, DAS/QU. (**d**) Raman spectra of DAS/ITO (green) and DAS/QU (red). Scale bar, 1 μm (**a**,**b**).

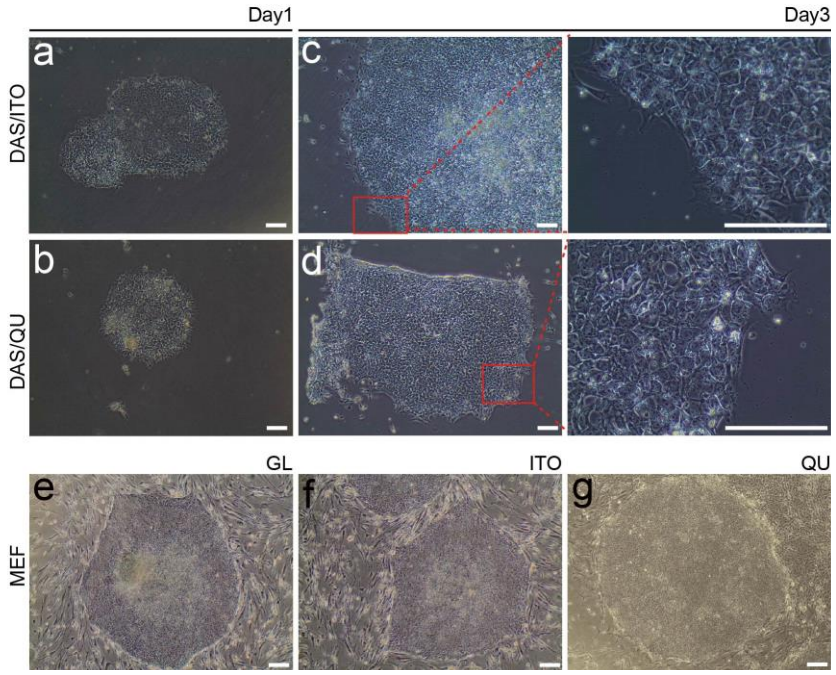

Figure S2. Morphology of hPSC on DAS/ITO, DAS/QU or MEF. (**a**,**b**) Morphology of hiPSC seeded on (**a**) DAS/ITO and (**b**) DAS/QU at day 1. (**c**,**d**) High magnification of hiPSC grown on (**c**) DAS/ITO and (**d**) DAS/QU at day 3. (**e**–**g**) Morphology of hiPSC co-cultured with MEF on (**e**) GL, (**f**) ITO and (**g**) QU at day 3. Scale bar, 200 μm (**a**–**g**).

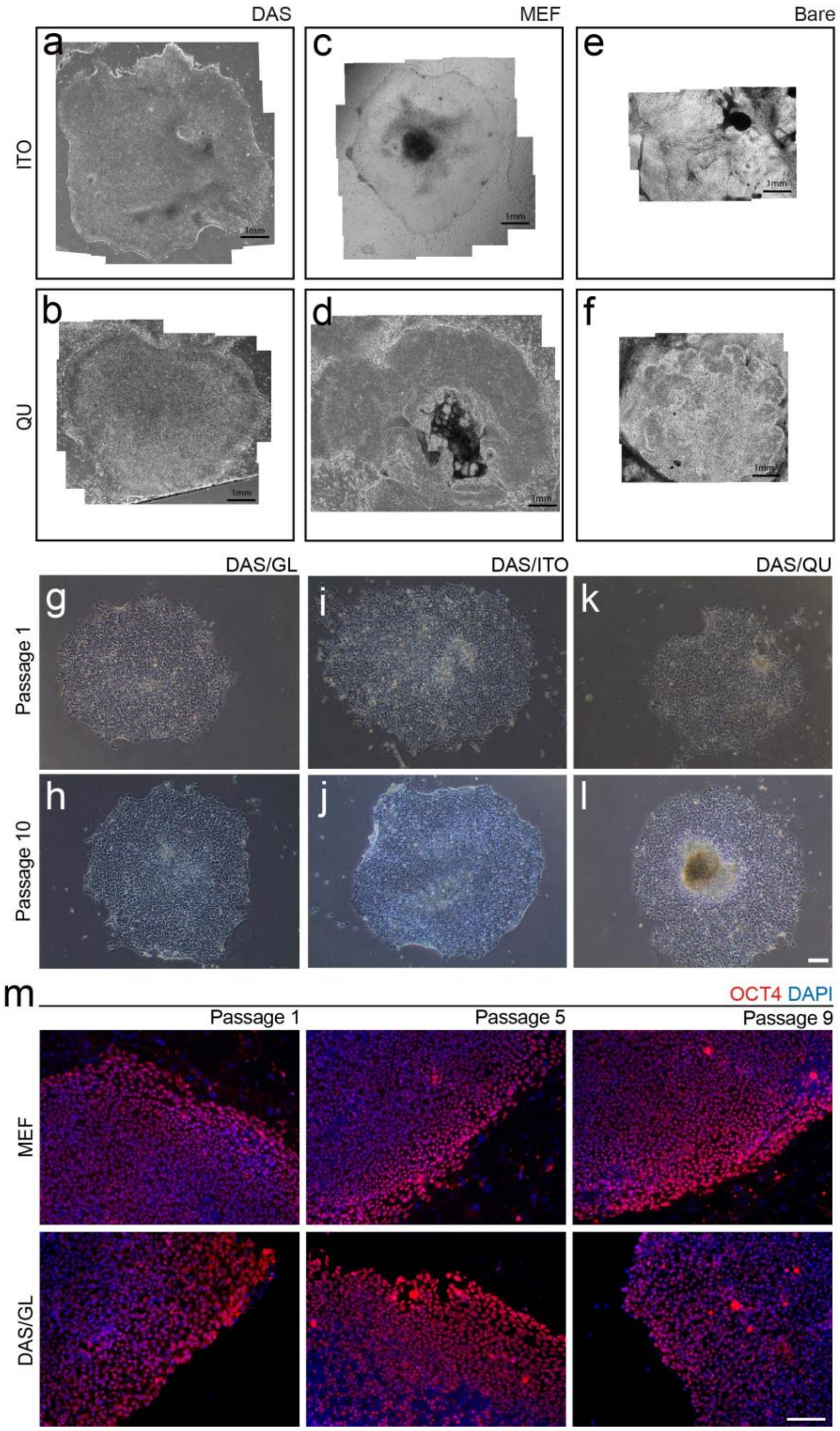

Figure S3. Long-term maintenance of undifferentiated hiPSC on DAS/GL, DAS/ITO or DAS/QU. (**a**-**f**) Morphology of hiPSC colony on (**a**) DAS/ITO and (**b**) DAS/QU or co-cultured with MEF on (**c**) ITO and (**d**) QU or on bare (**e**) ITO and (**f**) bare QU at 2 weeks of cultivation. (**g**–**j**) Morphology of hiPSC at passage 1 and passages 10 on (**g,h**) DAS/GL, (**i,j**) DAS/ITO and (**k,l**) DAS/QU. (**m**) Fluorescence images of $OCT4^+$ hiPSC cultured on MEF (top) or DAS/GL (bottom) at Passage 1 (P1), 5 (P5) and 9 (P9). Cells were counterstained with DAPI. Scale bar, 1µm (**a**–**f**), 200 µm (**g**–**l**), Scale bar, 150 µm (**m**).

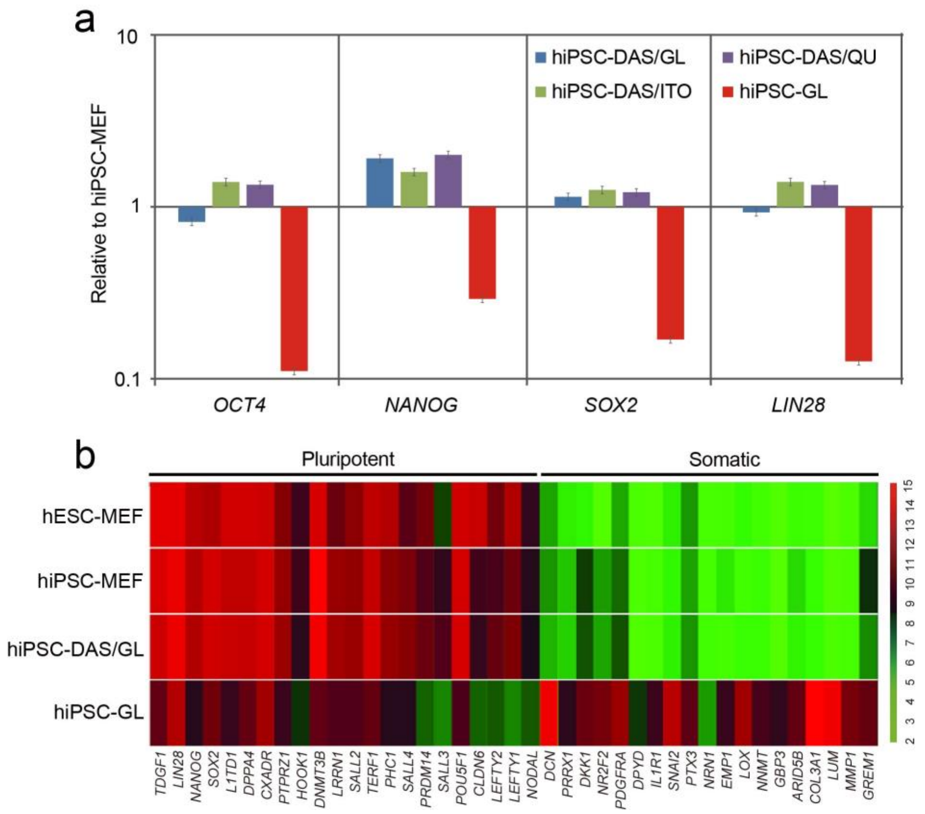

Figure S4. Molecular characterization of hiPSC cultured on DAS/NG. (**a**) Quantitative PCR (qRT-PCR) analysis for pluripotent markers (*OCT4*, *NANOG*, *SOX2* and *LIN28*) expression levels in hiPSC-DAS/GL, hiPSC-DAS/ITO, hiPSC-DAS/QU and hiPSC-GL relative to hiPSC-MEF. Transcript levels are normalized to *GAPDH* expression and represented in the logarithmic scale. Data are presented as mean ± s.e.m (n=3). (**b**) A heat map of microarray data showing gene expression of 22 pluripotent stem cell-enriched genes and 19 somatic cell-enriched genes within hESC-MEF, hiPSC-MEF, hiPSC-DAS/GL and hiPSC-GL. Color bar (right) indicates the color code gene expression in $\log_2$ scale.

Table S1. Average colony size of hiPSC and hESC on DAS-NG at day 14.

	Average colony size (mm)	
	hiPSC	hESC
DAS/GL	5.45 ± 0.10	5.12 ± 0.18
	4.87 ± 0.51	5.40 ± 0.11
DAS/ITO	4.52 ± 0.35	4.60 ± 0.41
	4.33 ± 0.41	4.33 ± 0.41
DAS/QU	4.36 ± 0.77	4.55 ± 0.59
	4.29 ± 0.30	3.94 ± 0.11

Data are shown as mean ± s.e.m (n=3).

Table S2. hPSC enriched cell adhesion gene expression of hiPSC or hESC grown on MEF, DAS/GL or bare GL.

	hESC-MEF	hiPSC-MEF	hiPSC-DAS/GL	hiPSC-GL	Expression enriched cell types
CDH9	7.0	6.7	7.1	4.0	hPSC
CELSR3	9.0	9.0	9.0	7.6	
CLDN6	9.8	9.7	9.5	7.3	
CLDN10	9.9	9.5	9.3	7.4	
MMP24	7.5	8.3	8.1	6.1	
RET	8.1	8.0	8.3	5.3	
CBLN1	7.3	7.9	8.6	5.3	hPSC and EB
CELSR2	8.6	8.4	8.4	7.3	
CDH4	7.1	6.7	7.1	5.8	
CDH6	7.9	9.1	9.2	8.8	
ITGB1	5.2	5.6	6.0	4.0	
ITGA6	8.8	8.7	9.4	8.2	
ITGA7	7.1	6.6	6.6	5.3	
LPHN1	9.4	9.3	9.2	7.9	
SORBS1	9.0	9.6	9.7	8.4	
SRF	9.1	9.0	9.1	8.2	
TIAM1	9.5	10.4	9.9	8.9	
DDR1	9.2	9.3	9.3	8.5	hPSC and EB
ITGA7	9.2	9.0	8.8	7.4	hPSC, EB and endoderm

The expression values in a log_2 scale obtained from numerical data of Heat Map are shown on Figure. 4b.

Table S3. Three-germ layer enriched cell adhesion gene expression of hiPSC or hESC grown on MEF, DAS/GL or bare GL.

	hESC-MEF	hiPSC-MEF	hiPSC-DAS/GL	hiPSC-GL	Expression enriched cell types
COL13A1	8.9	6.7	7.5	10.7	Mesoderm lineage
FBLN5	4.4	5.0	4.7	8.0	
PPFIBP2	7.7	6.4	7.3	9.3	
AGT	5.3	5.4	5.5	10.9	Endoderm lineage
ANGPTL3	4.1	4.0	4.5	8.0	
CEACAM1	4.9	4.6	4.4	6.8	
CLDN1	6.5	6.7	6.9	9.6	
CLDN15	8.1	8.0	8.1	9.0	
ECM2	3.9	4.1	4.2	8.1	
FGA	2.8	2.8	3.2	13.2	
FGG	4.6	4.9	4.8	14.0	
MPZL2	4.8	4.2	4.0	8.4	
VTN	5.3	5.5	5.6	10.9	
CDH19	3.5	3.4	3.5	6.2	Ectoderm lineage
L1CAM	7.6	8.3	8.0	9.7	
ATP2C1	7.5	6.9	6.8	7.8	Mesoderm and Ectoderm lineage
CD44	7.3	7.0	6.4	9.5	
CDH13	4.3	4.1	4.7	6.5	
ITGA4	6.5	5.5	5.7	8.0	
ITGA11	5.5	5.5	5.5	7.5	
ITGB1BP1	8.8	9.4	9.5	10.5	
PCDHGA1	7.9	8.1	8.0	9.0	
PPFIBP1	7.8	7.6	8.0	9.0	
CD164	8.0	8.8	8.5	9.2	All three germ layers
ITGA3	6.4	6.0	5.9	7.2	
CDH5	3.9	4.2	4.1	6.7	EB
CDH17	5.0	5.1	5.0	6.8	
FREM1	5.5	5.5	4.6	8.2	
NPNT	4.2	4.5	3.9	12.3	
PCDHA1	7.1	7.9	8.6	9.0	
PCDH17	6.3	6.7	5.5	9.0	
ITGB6	3.5	3.8	3.8	6.8	EB and Endoderm lineage
VCAM1	5.2	5.6	5.0	8.4	
CADM1	8.3	8.8	7.8	10.0	EB and Mesoderm lineage
COL3A1	5.8	3.9	4.1	13.9	
ITGA8	4.5	4.5	4.4	9.5	
NID2	8.2	8.0	7.3	12.6	
CDH7	5.1	4.8	5.1	6.3	EB and All three germ layers
CLSTN2	5.7	5.0	5.1	6.7	
EMP2	8.6	8.6	8.8	9.7	
PCDHB14	4.6	5.8	5.7	7.2	
PTPRM	7.7	6.8	6.6	10.8	
ITGAV	10.2	10.3	10.1	12.7	

The expression values in a log_2 scale obtained from numerical data of Heat Map are shown on Figure 4c.

Table S4. Quantitative real time PCR primers for cell-adhesion related genes.

Gene	Primer	Accession number
MEGF10	Forward: 5’-TGGTTTTTATGGGCATCGCT-3’<br>Reverse: 5’-TACAGGTTCCGTTGTTGGTG-3;	NM_032446.2
PCDH11X	Forward: 5’-CAGCGGAAATCTGAAGGGAA-3’<br>Reverse: 5’-GGGTGTAGCACGATCAAAGT-3’	NM_032968.4
COL1A2	Forward: 5’-CAGAGTGGAGCAGTGGTTAC-3’<br>Reverse: 5’-CAGTTCTTGGCTGGGATGTT-3’	NM_000089.3
HAPLN1	Forward: 5’-CGGAGTCAGGAACTACGGAT-3’<br>Reverse: 5’-CATAGGTCAGTTTGGTGGGG-3’	NM_001884.3

Table S5. Primary antibody list.

Antigen	Source	Isotype	Dilution
Oct3/4	Santacruz	Goat IgG	1:200
SSEA-4	Millipore	Mouse IgG3	1:200
TRA-1-60	Millipore	Mouse IgM	1:200
TRA-1-81	Millipore	Mouse IgM	1:200
TUJ1	Millipore	Mouse IgG1	1:200
α-SMA	Abcam	Mouse IgG2a	1:250
AFP	DAKO	Rabbit IgG	1:200
